# Risk factors associated with adverse outcomes after cervical conization in patients with cervical adenocarcinoma in situ

**DOI:** 10.1371/journal.pone.0325748

**Published:** 2025-06-09

**Authors:** Yuping Shan, Gulijinaiti Abulikemu, Lu Liu, Youzhong Zhang, Zhaoxia Ding

**Affiliations:** 1 Department of Obstetrics and Gynecology, The Affiliated Hospital of Qingdao University, Qingdao, China; 2 Department of Obstetrics and Gynecology, Qilu Hospital of Shandong University, Jinan, China; 3 Department of Gynecology, Qilu Hospital of Shandong University, Jinan, China; 4 Department of Gynecology, The Affiliated Hospital of Qingdao University, Qingdao, China; University of Brescia Department of Clinical and Experimental Sciences: Universita degli Studi di Brescia Dipartimento di Scienze Cliniche e Sperimentali, ITALY

## Abstract

**Background:**

Identifying high-risk groups for adverse outcomes after conization is crucial for developing targeted treatment plans for patients with cervical adenocarcinoma in situ (ACIS). This study aimed to analyze the clinical characteristics of patients with ACIS and identify risk factors associated with adverse outcomes.

**Methods:**

Patients diagnosed with ACIS through colposcopic biopsy at the Affiliated Hospital of Qingdao University and Qilu Hospital between January 2012 and December 2022 were selected. After meeting the inclusion and exclusion criteria, we collected their clinical data. Chi-square (χ^2^) tests and logistic regression models were employed to determine independent risk factors.

**Results:**

A total of 379 patients with ACIS were included in this analysis. About 26.1% of these patients tested positive on preoperative endocervical curettage (ECC), while 79.4% had a single lesion. Among the 334 patients who underwent cervical conization, 17.1% had positive surgical margins. Additionally, residual lesions were present in 53.6% of cases, and pathological upgrading occurred in 7.8% of patients. Multivariate analysis indicated that age (*p* < 0.001), preoperative histopathological results from ECC (*p* = 0.033), and the number of ACIS lesions (*p* < 0.001) were associated with positive surgical margins. Number of births (*p* = 0.011), preoperative histopathological results from ECC (*p* = 0.030), and surgical margin statuses at cervical conization (*p* < 0.001) were independent risk factors for residual lesions. Preoperative histopathological result of ECC (*p* = 0.035) was confirmed as a predictor of postoperative pathological upgrading.

**Conclusions:**

Older, multiparous patients with ACIS and abnormal preoperative ECC results require deeper diagnostic excision. Patients with positive conization margins necessitate further treatment, particularly when accompanied by abnormal ECC results. For women who wish to preserve their fertility, a repeat conization may be appropriate; however, in older and multiparous women, a hysterectomy would be recommended.

## Introduction

Cervical cancer (CC) is the fourth leading cause of cancer death among women worldwide, resulting in over 500,000 new cases and 300,000 deaths annually [1]. Histologically, cervical squamous cell carcinoma (CSCC) constitutes approximately 75% of all CC cases, whereas cervical adenocarcinoma (CAC) accounts for about 25% [2,3]. Despite the decline in CSCC incidence due to advances in cervical cytology, HPV testing, and colposcopy, a similar reduction has not been observed in CAC. Characterized by its aggressive phenotype and resistance to radiotherapy, the lack of a decrease in CAC incidence is primarily due to its delayed diagnosis, representing a significant health concern for women [4,5].

In 1952, Hepler et al. first described the precursor to CAC, known as cervical adenocarcinoma in situ (ACIS), which involves the replacement of some or all cervical glands by atypical glandular epithelium [6]. Over recent decades, the incidence of ACIS—which may coexist with CAC and squamous dysplasia—has risen, particularly among individuals aged 30–40 years [7]. Persistent infection with high-risk human papillomavirus (hrHPV), especially types 16 and 18, is widely recognized as the primary cause of ACIS [8]. Typically, ACIS originates in the transformation zone of the cervix but can also extend higher into the endocervical canal, affecting the deeper parts of the endocervical clefts [9]. At present, it is well recognized that timely detection and treatment of cervical ACIS can reduce the incidence of CAC and improve patient prognosis, as the typical interval between clinically detectable ACIS and early invasion is at least five years [10]. The American Society for Colposcopy and Cervical Pathology (ASCCP) Risk-Based Management Consensus Guidelines recommend cervical cytology, HPV testing, cervical biopsy, and endocervical curettage (ECC) under colposcopy for evaluating suspected ACIS cases [4,11]. However, diagnosing and treating ACIS presents certain challenges due to its atypical clinical presentations, multifocality, and the low sensitivity of screening approaches. Consequently, a diagnostic excisional procedure is advised for all patients with a histological diagnosis of ACIS [11]. Cold knife conization (CKC) and loop electrosurgical excision procedure (LEEP) are traditionally recommended to confirm diagnosis, exclude CAC, and assess margin status [12]. Notably, while hysterectomy is advised for patients with ACIS following diagnostic conization, conization alone is considered acceptable for those desiring pregnancy, provided the margins are negative, and they meet surveillance standards [13]. Identifying high-risk groups for adverse outcomes after conization is critical for developing targeted treatment plans for ACIS patients and has become a focal point in medical research. However, the factors influencing postoperative outcomes for ACIS patients remain poorly understood, which may affect prognosis judgments and treatment decisions.

In this study, we analyzed the clinical characteristics of patients with ACIS at the Affiliated Hospital of Qingdao University and Qilu Hospital of Shandong University to explore the risk factors associated with adverse outcomes, including positive surgical margins, residual lesions and pathological upgrading after cervical conization. This study aims to evaluate the safety of cervical conization in ACIS patients, predict their prognosis, and aid in determining individualized treatment options.

## Materials and methods

This is a retrospective study based on data obtained from a network of the Affiliated Hospital of Qingdao University and Qilu Hospital of Shandong University. First, we selected patients diagnosed with ACIS through colposcopic biopsy and screened them based on inclusion and exclusion criteria. Subsequently, we utilized the hospital network to gather clinical characteristics, surgical information, and follow-up data for the patients ultimately included in the study. Finally, we classified and analyzed the collected data.

All procedures involving human participants were conducted in accordance with the ethical standards of the institutional and/or national research committee, the 1964 Helsinki Declaration, and its subsequent amendments or comparable ethical standards. The study received approval from the ethics committee of the Affiliated Hospital of Qingdao University (approval number: QYFY WZLL27495) on December 13, 2022. Written informed consent was obtained from patients upon their arrival at the study hospital for treatment. For minors, consent was obtained from parents or guardians. Data were accessed for research purposes on January 1, 2023.

### Study population selection

We retrospectively selected patients diagnosed with ACIS via colposcopic biopsy at the Affiliated Hospital of Qingdao University and Qilu Hospital of Shandong University between January 2012 and December 2022. We subsequently screened these patients based on the inclusion and exclusion criteria. Inclusion criteria: The patient was initially treated at the Affiliated Hospital of Qingdao University or Qilu Hospital of Shandong University. Exclusion criteria: (1) Patients who underwent hysterectomy for other gynecologic malignancies, where ACIS was identified postoperatively; (2) Patients who were lost to follow-up or lacked follow-up information regarding cervical lesions.

### Clinical information

After screening the included patients, we retrospectively collected the following clinical data from the hospital network: age, number of pregnancies, number of births, clinical symptoms, forms of contraception, preoperative HPV testing results, preoperative cervical cytology results, preoperative transformation zone (TZ) types observed under colposcopy, preoperative diagnoses from colposcopy (clinical colposcopy impression), preoperative histopathological results from ECC performed during colposcopy, the number of ACIS lesions (pathological biopsy under initial colposcopy). Normally, these patients underwent cervical conization or hysterectomy after being diagnosed with ACIS through colposcopic biopsy. Consequently, we can utilize the hospital network to collect not only the patients’ clinical characteristics but also their surgical information. This includes intraoperative surgical margin statuses, postoperative pathological diagnoses, and postoperative immunohistochemical expressions of Ki-67. Except for a few patients with positive surgical margins who directly underwent a second surgery within six months after the initial conization, the remaining patients with positive surgical margins, as well as those with negative surgical margins, received the same follow-up plan. According to the recommendations of the Society of Gynecologic Oncology (SGO), these patients were routinely monitored through cervical cytology and HPV testing following surgery. If the results of cervical cytology and HPV testing were normal, patients can be classified as normal following surgery. If the results of cervical cytology and HPV testing were abnormal, they underwent a colposcopic biopsy for further examination. During this process, some patients had significant follow-up information missing due to reasons such as seeking care at other hospitals. Consequently, we did not follow up with these patients, and they were excluded from this study. This process allowed us to retrospectively collect their postoperative follow-up data.

The outer edge of resection for both CKC and LEEP was set at 0.5 cm from the lesion, with a resection height ranging from 1.5 to 2.5 cm. In this study, adverse outcomes following cervical conization in patients with ACIS included positive surgical margins, residual lesions, and pathological upgrading. The evaluation criteria for adverse outcomes after cervical conization were as follows: (1) Positive surgical margins: The presence of ACIS was in the first conization surgical margins or within 1 mm of these margins, regardless of whether they were ecto-, endo-, or stromal margins, which is consistent with previous studies [14]. If patients had multiple tissue specimens removed during a single conization excision, a positive surgical margin is indicated when the surgical margin in the final tissue specimen is positive. (2) Residual lesions: High-grade squamous intraepithelial lesions (HSIL) or ACIS detected in a cervical biopsy during colposcopy within six months of the initial conization, or identified in the histopathology from a second surgery (conization/hysterectomy) within six months of the initial conization, were classified as residual lesions. The similar etiology and treatment approaches of ACIS and HSIL justify their inclusion as adverse outcomes in this analysis, noting that some patients present with both conditions. (3) Postoperative pathological upgrading: The detection of CC in a cervical biopsy during colposcopy within six months after the initial conization, or in the histopathology from a second surgery (conization/hysterectomy) within six months after the initial conization, was classified as pathological upgrading.

SurePath™ liquid-based Pap test (BD, USA), an automated liquid-based cytology system, was utilized for all cytologic analyses. All cytological smears were evaluated by an experienced cytopathologist following the 2001 Bethesda System. This includes categories such as negative for intraepithelial lesion and malignancy (NILM), atypical squamous cells of undetermined significance (ASC-US), low-grade squamous intraepithelial lesion (LSIL), atypical squamous cells that cannot exclude HSIL (ASC-H), HSIL, atypical glandular cells (AGC), and invasive CC. HPV DNA genotype testing was conducted using the YaNeng PCR-RDB HPV genotyping kit (Yaneng Biosciences, Shenzhen, China), capable of detecting 23 HPV genotypes. This includes hrHPV types (16, 18, 31, 33, 35, 39, 45, 51, 52, 53, 56, 58, 59, 66, 68, 73, and 82) and low-risk HPV (lrHPV) types (82, 83, 6, 11, 42, 43, and 81). Cervical biopsies under colposcopy and ECC were performed by two expert colposcopists in accordance with standard protocols, with results interpreted according to the International Federation for Cervical Pathology and Colposcopy (IFCPC) nomenclature. Specifically, a type 1 TZ (TZ 1) is entirely ectocervical and fully visible; a type 2 TZ (TZ 2) includes part or all of the squamocolumnar junction (SCJ) within the endocervical canal but remains fully visible; and a type 3 TZ (TZ 3) also includes the SCJ within the endocervical canal but is not fully visible. According to the ASCCP, clinical colposcopy impressions are classified into six categories: NILM, LSIL, HSIL, suspected CC, suspected AGC, and other findings. Therefore, in our study, we categorized preoperative colposcopy diagnoses (clinical colposcopy impression) based on HSIL. Multiple ACIS lesions are defined as two or more distinctly distributed lesions within normal cervical epithelial tissue observed during the pathological biopsy under initial colposcopy. If a patient exhibited multiple pathological grades before surgery, the highest preoperative pathological grade was considered, consistent with the postoperative pathological grade.

### Statistics analysis

Variables were described using means ± standard deviations (SD) or numbers (percentages). Dichotomization cutoffs for logistic regression were based on previous research by Kietpeerakool et al. [15]. Comparisons between different groups were conducted using the chi-square (χ²) test, and factors found to be statistically significant were included in multivariate analyses using logistic regression models. Results are presented as odds ratios (OR) with 95% confidence intervals (CI). All statistical analyses were performed using SPSS software (version 29.0; IBM Corp., USA). A probability (*p*)-value of <0.05 was considered statistically significant.

## Results

**Fig 1:**
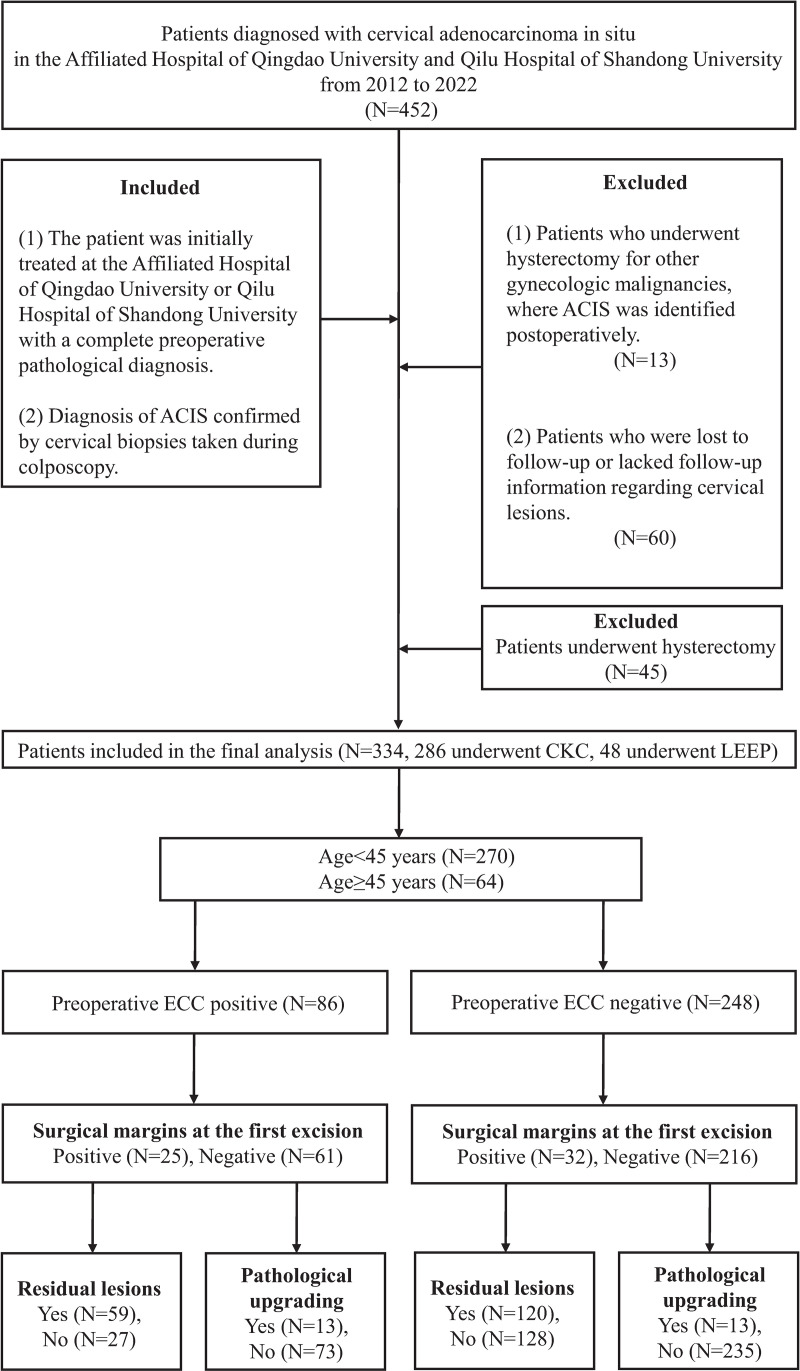
Flowchart of the study selection.

A total of 452 patients diagnosed with ACIS were identified at the Affiliated Hospital of Qingdao University and Qilu Hospital of Shandong University between January 2012 and December 2022. Following the inclusion and exclusion criteria, 379 patients were included in the final analysis (Fig 1).

### Characteristics of the patients

Baseline characteristics of the selected patients are presented in Table 1. The mean age of the entire group was 39.5 ± 8.2 years, ranging from 17 to 70 years. Among these, 41.4% were between 30 and 40 years old. On average, the number of pregnancies and births were 2.6 and 1.3, respectively. Approximately 13.7% of the patients exhibited contact bleeding, while the remaining 86.3% had no apparent clinical symptoms or presented with other symptoms. Regarding contraception, 41.7% of the patients used barrier methods, compared to 58.3% who used non-barrier methods or no contraception at all. Additionally, a total of 374 patients were tested for HPV before surgery, with 90.0% testing positive for hrHPV. Of the 324 patients who underwent preoperative cervical cytological examination, 31.7% had results milder than ASC-US. Information regarding preoperative TZ types under colposcopy was missing for 88 patients; 38.0% had a TZ 1, while 38.8% had HSIL or more severe lesions. Moreover, 26.1% of patients tested positive in ECC. Regarding the number of ACIS lesions, 79.4% presented with a single lesion. Notably, out of 379 patients, 45 underwent a direct hysterectomy, 286 received CKC, and 48 received LEEP. Patients who had undergone hysterectomy were excluded from the subsequent analyses of adverse outcomes following cervical conization. There were 45 patients with unknown postoperative pathological diagnoses, some of whom had a direct hysterectomy. About 10.0% were diagnosed postoperatively with ACIS coexisting with HSIL. Additionally, 29.0% of patients exhibited a Ki-67 positivity rate of 70% or higher. Among the 334 patients who underwent CKC or LEEP, 57 had positive surgical margins, 179 had residual lesions (169 with ACIS, 1 with ACIS+HSIL, 9 with HSIL), and 26 experienced postoperative pathological upgrading. During our follow-up, 32 of the 57 patients with positive surgical margins underwent second surgery. The pathological results from this secondary procedure indicated that 29 patients were HSIL/ACIS, 2 patients were CC, and 1 patient was found to be normal. Additionally, 25 patients who did not undergo second surgery received cervical cytology and HPV testing, along with 277 patients who had negative surgical margins. Among these, 116 patients had normal results of cervical cytology and HPV testing, while 186 patients underwent colposcopic biopsy due to abnormal findings. The results of the colposcopic pathological biopsies revealed that 24 patients were CC, 150 patients were HSIL/ACIS, and 12 patients were normal.

**Table 1:** Clinical characteristics of patients with ACIS by preoperative pathological diagnosis in this study. (N = 379).

Characteristics	mean ± SD or n (%)
Age (years)	39.5 ± 8.2
Number of pregnancies (times)	2.6 ± 1.5
Number of births (times)	1.3 ± 0.7
Clinical symptoms	
Unapparent clinical symptoms	252 (66.5%)
Contact bleeding	52 (13.7%)
Irregular vaginal bleeding	38 (10.0%)
Increased vaginal secretion	19 (5.0%)
Abnormal vaginal discharge	8 (2.1%)
Abdominal pain	10 (2.6%)
Forms of contraception	
Barrier contraception	158 (41.7%)
Non-barrier contraception or no contraception	221 (58.3%)
Preoperative cervical cytology results	
NILM	120 (31.7%)
ASC-US	56 (14.8%)
LSIL	19 (5.0%)
ASC-H	31 (8.2%)
HSIL	61 (16.1%)
AGC	35 (9.2%)
Abnormal results (details unknown)	2 (0.5%)
NA	55 (14.5%)
Preoperative HPV testing results	
Positive for hrHPV	341 (90.0%)
Negative for hrHPV	33 (8.7%)
NA	5 (1.3%)
Preoperative TZ types under colposcopy	
TZ 1	144 (38.0%)
TZ 2	79 (20.8%)
TZ 3	68 (17.9%)
NA	88 (23.2%)
Preoperative diagnoses under colposcopy (clinical colposcopy impression)	
Normal	19 (5.0%)
LSIL	125 (33.0%)
HSIL	139 (36.7%)
Suspected CC	8 (2.1%)
NA	88 (23.2%)
Preoperative histopathological results of ECC	
Positive	99 (26.1%)
Negative	280 (73.9%)
Number of ACIS lesions (pathological biopsy under initial colposcopy)	
≥2	78 (20.6%)
1	301 (79.4%)
Postoperative pathological diagnosis (initial surgery)	
ACIS+HSIL	38 (10.0%)
ACIS	296 (78.1%)
NA	45 (11.9%)
Postoperative immunohistochemical expressions of Ki-67	
≥70%	110 (29.0%)
<70%	125 (33.0%)
NA	144 (38.0%)
The procedure for cervical conization^*^	
CKC	286 (85.6%)
LEEP	48 (14.4%)
Surgical margins at cervical conization^*^	
Positive	57 (17.1%)
Negative	277 (82.9%)
Residual lesions after cervical conization^*^	
Yes	179 (53.6%)
No	155 (46.4%)
Postoperative pathological upgrading at/after cervical conization^*^	
Yes	26 (7.8%)
No	308 (92.2%)

* The patients who underwent hysterectomy (n=45) were excluded.

ACIS, cervical adenocarcinoma in situ; AGC, atypical glandular cells; ASC-H, atypical squamous cells cannot exclude high-grade squamous intraepithelial lesions; ASC-US, atypical squamous cells of undetermined significance; CC, cervical cancer; CKC, cold knife conization; ECC, endocervical curettage; hrHPV, high-risk human papillomavirus; HSIL, high-grade squamous intraepithelial lesions; LEEP, loop electrosurgical excision procedure; LSIL, low-grade squamous intraepithelial lesion; NILM, negative for intraepithelial lesion and malignancy; SD, standard deviations; TZ, transformation zone. NA means missing data.

### Risk factors for positive surgical margins

A total of 334 patients who underwent either CKC or LEEP were categorized into two groups: those with positive surgical margins and those with negative surgical margins. Table 2 displays significant differences between these groups in terms of age (*p* < 0.001), preoperative diagnoses under colposcopy (*p* < 0.001), preoperative histopathological results of ECC (*p* < 0.001), number of ACIS lesions (*p* < 0.001), and postoperative immunohistochemical expressions of Ki-67 (*p* < 0.001). Due to substantial data loss concerning preoperative diagnoses under colposcopy and postoperative immunohistochemical expressions of Ki-67, these variables were excluded from the multivariate analysis. This considerable amount of missing data could potentially affect the results of the analysis. Multivariate analyses revealed that age (≥45 *vs.* < 45 years, OR=3.660, 95% CI: 1.696–7.898, *p* < 0.001), positive preoperative histopathological results of ECC (Positive *vs.* Negative, OR=2.247, 95% CI: 1.066–4.736, *p* = 0.033), and number of ACIS lesions (≥2 *vs*. < 2, OR=18.961, 95% CI: 8.765–41.018, *p* < 0.001) were independent risk factors for positive surgical margins at cervical conization in patients with ACIS (Table 2). Subsequently, we summarized the depth of the first conization and the surgical margin status of these three groups, as shown in Table 3. We found that patients aged 45 years and older, those with positive preoperative histopathological ECC results, and individuals with multiple lesions exhibited higher rates of positive surgical margins. However, their mean depth of the first conization was greater. This may suggest that in our study, doctors performed deeper resections in these patients, however, the depth may not have been sufficient. Therefore, when conization is performed on these patients, the depth specified in Table 2 should be exceeded.

**Table 2:** Risk factors for positive surgical margins at cervical conization in patients with ACIS (N = 334, treated with CKC/LEEP, referring to the initial surgery results).

Characteristics	n (%)	With positive surgical margins (n = 57)	With negative surgical margins (n = 277)	Univariate analysis		Multivariate analysis	
				OR (95% CI)	*p*-value	OR (95% CI)	*p*-value
Age (years)				4.768 (2.557, 8.891)	<0.001	3.660 (1.696, 7.898)	<0.001
≥45	64 (19.2%)	25 (43.9%)	39 (14.1%)				
<45	270 (80.8%)	32 (56.1%)	238 (85.9%)				
Number of pregnancies (times)				1.839 (0.967, 3,497)	0.061	–	–
≥4	69 (20.7%)	17 (29.8%)	52 (18.8%)				
<4	265 (79.3%)	40 (70.2%)	225 (81.2%)				
Number of births (times)				1.450 (0.813, 2.585)	0.207	–	–
≥2	122 (36.5%)	25 (43.9%)	97 (35.0%)				
<2	212 (63.5%)	32 (56.1%)	180 (65.0%)				
Clinical symptoms				0.694 (0.332, 1.450)	0.329	–	–
Contact bleeding	75 (22.5%)	10 (17.5%)	65 (23.5%)				
Others	259 (77.5%)	47 (82.5%)	212 (76.5%)				
Forms of contraception				1.005 (0.564, 1.791)	0.985	–	–
Non-barrier contraception or no contraception	193 (57.8%)	33 (57.9%)	160 (57.8%)				
Barrier contraception	141 (42.2%)	24 (42.1%)	117 (42.2%)				
Preoperative HPV testing results				1.272 (0.424, 3.818)	0.667	–	–
Positive for hrHPV	303 (90.7%)	53 (93.0%)	250 (91.2%)				
Negative for hrHPV	28 (8.4%)	4 (7.0%)	24 (8.8%)				
NA	3 (0.9%)	0	3				
Preoperative cervical cytology results				1.590 (0.792, 3.189)	0.189	–	–
≥ASC-US	178 (53.3%)	31 (70.5%)	147 (60.0%)				
<ASC-US	111 (33.2%)	13 (29.5%)	98 (40.0%)				
NA	45 (13.5%)	13	32				
Preoperative TZ types under colposcopy				1.434 (0.745, 2.762)	0.279	–	–
TZ 2 or 3	125 (37.4%)	25 (56.8%)	100 (47.8%)				
TZ 1	128 (38.3%)	19 (43.2%)	109 (52.2%)				
NA	81 (24.3%)	13	68				
Preoperative diagnoses under colposcopy (clinical colposcopy impression)				11.771 (4.065, 34.084)	<0.001	–	–
≥HSIL	136 (40.7%)	40 (90.0%)	96 (45.9%)				
<HSIL	117 (35.0%)	4 (9.1%)	113 (54.1%)				
NA	81 (24.3%)	13	68				
Preoperative histopathological results of ECC				2.766 (1.525, 5.017)	<0.001	2.247 (1.066, 4.736)	0.033
Positive	86 (25.7%)	25 (43.9%)	61 (22.0%)				
Negative	248 (74.3%)	32 (56.1%)	216 (78.0%)				
Number of ACIS lesions (pathological biopsy under initial colposcopy)				20.880 (10.093, 43.197)	<0.001	18.961 (8.765, 41.018)	<0.001
≥2	48 (14.4%)	32 (56.1%)	16 (5.8%)				
1	286 (85.6%)	25 (43.9%)	261 (94.2%)				
The procedure for cervical conization				0.560 (0.271, 1.159)	0.118	–	–
CKC	286 (85.6%)	45 (78.9%)	241 (87.0%)				
LEEP	48 (14.4%)	12 (21.1%)	36 (13.0%)				
Postoperative immunohistochemical expressions of Ki-67				7.730 (3.596, 16.619)	<0.001	–	–
≥70%	95 (28.4%)	39 (79.6%)	56 (33.5%)				
<70%	121 (36.2%)	10 (20.4%)	111 (66.5%)				
NA	118 (35.3%)	8	110				

ACIS, cervical adenocarcinoma in situ; AGC, atypical glandular cells; ASC-H, atypical squamous cells cannot exclude high-grade squamous intraepithelial lesions; ASC-US, atypical squamous cells of undetermined significance; CC, cervical cancer; CKC, cold knife conization; ECC, endocervical curettage; hrHPV, high-risk human papillomavirus; HSIL, high-grade squamous intraepithelial lesions; LEEP, loop electrosurgical excision procedure; LSIL, low-grade squamous intraepithelial lesion; NILM, negative for intraepithelial lesion and malignancy; SD, standard deviations; TZ, transformation zone. NA means missing data.

**Table 3:** The depth of first conization and surgical margin status in different groups.

Characteristics	The depth of first conization (mean ± SD, cm)	n (%)	With positive surgical margins (n = 57)	With negative surgical margins (n = 277)
Age (years)				
≥45	2.1 ± 0.2	64 (19.2%)	25 (39.1%)	39 (60.9%)
<45	2.0 ± 0.3	270 (80.8%)	32 (11.9%)	238 (88.1%)
Preoperative histopathological results of ECC				
Positive	2.2 ± 0.4	86 (25.7%)	25 (29.1%)	61 (70.9%)
Negative	1.9 ± 0.2	248 (74.3%)	32 (12.9%)	216 (87.1%)
Number of ACIS lesions (pathological biopsy under initial colposcopy)				
≥2	2.2 ± 0.2	48 (14.4%)	32 (66.7%)	16 (33.3%)
1	1.8 ± 0.3	286 (85.6%)	25 (8.7%)	261 (91.3%)

ACIS, cervical adenocarcinoma in situ; ECC, endocervical curettage; SD, standard deviations.

### Risk factors for residual lesions

A total of 334 patients who underwent cervical conization were divided into two groups based on the presence of residual lesions: those with residual lesions and those without. Univariate analyses revealed significant associations between residual lesions and several factors: age (*p* = 0.032), number of births (*p* = 0.002), forms of contraception (*p* = 0.019), preoperative histopathological results of ECC (*p* = 0.001), number of ACIS lesions (*p* = 0.004), and surgical margin statuses at cervical conization (*p* < 0.001) (Table 4). Multivariate analyses, also presented in Table 4, identified the number of births (≥2 *vs*. < 2, OR=1.904, 95% CI: 1.158–3.131, *p* = 0.011), positive preoperative histopathological results of ECC (Positive *vs*. Negative, OR=1.859, 95% CI: 1.063–3.252, *p* = 0.030), and surgical margin statuses at cervical conization (Positive *vs*. Negative, OR=6.954, 95% CI: 2.905–16.645, *p* < 0.001) as independent risk factors for residual lesions.

**Table 4:** Risk factors for residual lesions after cervical conization in patients with ACIS (N = 334, treated with CKC/LEEP).

Characteristics	n (%)	With residual lesions (n = 179)	Without residual lesions (n = 155)	Univariate analysis		Multivariate analysis	
				OR (95% CI)	*p*-value	OR (95% CI)	*p*-value
Age (years)				1.853 (1.050, 3.272)	0.032	0.854 (0.432, 1.689)	0.651
≥45	64 (19.2%)	42 (23.5%)	22 (14.2%)				
<45	270 (80.8%)	137 (76.5%)	133 (85.8%)				
Number of pregnancies (times)				1.695 (0.981, 2.982)	0.057	–	–
≥4	69 (20.7%)	44 (24.6%)	25 (16.1%)				
<4	265 (79.3%)	135 (75.4%)	130 (83.9%)				
Number of births (times)				2.058 (1.300, 3.256)	0.002	1.904 (1.158, 3.131)	0.011
≥2	122 (36.5%)	79 (44.1%)	43 (27.7%)				
<2	212 (63.5%)	100 (55.9%)	112 (72.3%)				
Clinical symptoms				1.057 (0.631, 1.771)	0.832	–	–
Contact bleeding	75 (22.5%)	41 (22.9%)	34 (21.9%)				
Others	259 (77.5%)	138 (77.1%)	121 (78.1%)				
Forms of contraception				1.687 (1.089, 2.615)	0.019	1.605 (0.986, 2.610)	0.057
Non-barrier contraception or no contraception	193 (57.8%)	114 (63.7%)	79 (51.0%)				
Barrier contraception	141 (42.2%)	65 (36.3%)	76 (49.0%)				
Preoperative HPV testing results				2.144 (0.958, 4.800)	0.059	–	–
Positive for hrHPV	303 (90.7%)	162 (94.2%)	136 (88.35%)				
Negative for hrHPV	28 (8.4%)	10 (5.8%)	18 (11.7%)				
NA	3 (0.9%)	2	1				
Preoperative cervical cytology results				1.390 (0.864, 2.236)	0.174	–	–
≥ASC-US	178 (53.3%)	98 (65.3%)	80 (57.6%)				
<ASC-US	111 (33.2%)	52 (34.7%)	59 (42.4%)				
NA	45 (13.5%)	29	16				
Preoperative TZ types under colposcopy				1.409 (0.856, 2.319)	0.177	–	–
TZ 2 or 3	125 (37.4%)	75 (53.2%)	50 (44.6%)				
TZ 1	128 (38.3%)	66 (46.8%)	62 (55.4%)				
NA	81 (24.3%)	38	43				
Preoperative diagnoses under colposcopy (clinical colposcopy impression)				1.420 (0.862, 2.338)	0.167	–	–
≥HSIL	136 (40.7%)	74 (52.5%)	49 (43.8%)				
<HSIL	117 (35.0%)	67 (47.5%)	63 (56.2%)				
NA	81 (24.3%)	38	43				
Preoperative histopathological results of ECC				2.331 (1.387, 3.917)	0.001	1.859 (1.063, 3.252)	0.030
Positive	86 (25.7%)	59 (33.0%)	27 (17.4%)				
Negative	248 (74.3%)	120 (67.0%)	128 (82.6%)				
Number of ACIS lesions (pathological biopsy under initial colposcopy)				2.655 (1.348, 5.227)	0.004	1.497 (0.689, 3.251)	0.308
≥2	48 (14.4%)	35 (19.6%)	13 (8.4%)				
1	286 (85.6%)	14 (80.4%)	261 (94.2%)				
The procedure for cervical conization				0.795 (0.453, 1.211)	0.145	–	–
CKC	286 (85.6%)	148 (82.7%)	138 (89.0%)				
LEEP	48 (14.4%)	31 (17.3%)	17 (11.0%)				
Surgical margin statuses at cervical conization				8.195 (3.590, 18.708)	<0.001	6.954 (2.905, 16.645)	<0.001
Positive	57 (17.1%)	50 (27.9%)	7 (4.5%)				
Negative	277 (82.9%)	129 (72.1%)	148 (95.5%)				
Postoperative immunohistochemical expressions of Ki-67				1.534 (0.186, 2.656)	0.126	–	–
≥70%	95 (28.4%)	53 (51.0%)	42 (40.4%)				
<70%	121 (36.2%)	51 (49.0%)	62 (59.6%)				
NA	118 (35.3%)	75	51				

ACIS, cervical adenocarcinoma in situ; AGC, atypical glandular cells; ASC-H, atypical squamous cells cannot exclude high-grade squamous intraepithelial lesions; ASC-US, atypical squamous cells of undetermined significance; CC, cervical cancer; CKC, cold knife conization; ECC, endocervical curettage; hrHPV, high-risk human papillomavirus; HSIL, high-grade squamous intraepithelial lesions; LEEP, loop electrosurgical excision procedure; LSIL, low-grade squamous intraepithelial lesion; NILM, negative for intraepithelial lesion and malignancy; SD, standard deviations; TZ, transformation zone. NA means missing data.

### Risk factors for postoperative pathological upgrading

In our study, 334 patients who underwent conization were divided into two groups: those with postoperative pathological upgrading and those without. Table 5 shows that age (*p* = 0.037), preoperative histopathological results of ECC (*p* = 0.003), number of ACIS lesions (*p* = 0.013), surgical margin statuses at cervical conization (*p* < 0.001), and postoperative immunohistochemical expressions of Ki-67 (*p* = 0.005) were associated with postoperative pathological upgrading. Due to a significant number of missing data points for postoperative immunohistochemical expressions of Ki-67, this variable was excluded from the multivariate analysis. Additionally, preoperative histopathological results of ECC (Positive *vs*. Negative, OR=2.502, 95% CI: 0.572–4.605, *p* = 0.035) were identified as an independent risk factor for postoperative pathological upgrading in our study (Table 5).

**Table 5:** Risk factors for postoperative pathological upgrading at/after cervical conization in patients with ACIS (N = 334, treated with CKC/LEEP).

Characteristics	n (%)	With postoperative pathological upgrading (n = 26)	Without postoperative pathological upgrading (n = 308)	Univariate analysis		Multivariate analysis	
				OR (95% CI)	*p*-value	OR (95% CI)	*p*-value
Age (years)				2.435 (1.032, 5.749)	0.037	1.336 (0.510, 3.501)	0.556
≥45	64 (19.2%)	9 (34.6%)	55 (17.9%)				
<45	270 (80.8%)	17 (65.4%)	253 (82.1%)				
Number of pregnancies (times)				1.800 (0.747, 4.333)	0.185	–	–
≥4	69 (20.7%)	8 (30.8%)	61 (19.8%)				
<4	265 (79.3%)	18 (69.2%)	247 (80.2%)				
Number of births (times)				1.826 (0.818, 4.077)	0.137	–	–
≥2	122 (36.5%)	13 (50.0%)	109 (35.4%)				
<2	212 (63.5%)	13 (50.0%)	199 (64.6%)				
Clinical symptoms				1.599 (0.666, 3.838)	0.290	–	–
Contact bleeding	75 (22.5%)	8 (30.8%)	67 (21.8%)				
Others	259 (77.5%)	18 (69.2%)	241 (78.2%)				
Forms of contraception				1.184 (0.521, 2.693)	0.687	–	–
Non-barrier contraception or no contraception	193 (57.8%)	16 (61.5%)	177 (57.5%)				
Barrier contraception	141 (42.2%)	10 (38.5%)	131 (42.5%)				
Preoperative HPV testing results				2.323 (0.302, 17.844)	0.405	–	–
Positive for hrHPV	303 (90.7%)	24 (96.0%)	279 (91.2%)				
Negative for hrHPV	28 (8.4%)	1 (4.0%)	27 (8.8%)				
NA	3 (0.9%)	1	2				
Preoperative cervical cytology results				1.367 (0.539, 3.466)	0.508	–	–
≥ASC-US	178 (53.3%)	15 (68.2%)	163 (61.0%)				
<ASC-US	111 (33.2%)	7 (31.8%)	104 (39.0%)				
NA	45 (13.5%)	4	41				
Preoperative TZ types under colposcopy				1.214 (0.486, 3.034)	0.678	–	–
TZ 2 or 3	125 (37.4%)	14 (63.6%)	111 (59.0%)				
TZ 1	128 (38.3%)	8 (36.4%)	120 (41.0%)				
NA	81 (24.3%)	4	77				
Preoperative diagnoses under colposcopy (clinical colposcopy impression)				1.959 (0.791, 4.848)	0.140	–	–
≥HSIL	136 (40.7%)	14 (63.6%)	109 (47.2%)				
<HSIL	117 (35.0%)	8 (36.4%)	122 (52.8%)				
NA	81 (24.3%)	4	77				
Preoperative histopathological results of ECC				3.219 (1.429, 7.254)	0.003	2.502 (0.572, 4.605)	0.035
Positive	86 (25.7%)	13 (50.0%)	73 (23.7%)				
Negative	248 (74.3%)	13 (50.0%)	235 (76.3%)				
Number of ACIS lesions (pathological biopsy under initial colposcopy)				2.978 (1.215, 7.300)	0.013	1.623 (1.064, 5.881)	0.363
≥2	48 (14.4%)	8 (30.8%)	40 (13.0%)				
1	286 (85.6%)	18 (69.2%)	268 (87.0%)				
The procedure for cervical conization				0.714 (0.011, 1.081)	0.250	–	–
CKC	286 (85.6%)	6 (23.1%)	280 (90.9%)				
LEEP	48 (14.4%)	20 (76.9%)	28 (9.1%)				
Surgical margin statuses at cervical conization				4.177 (1.805, 9.663)	<0.001	2.573 (0.933, 7.098)	0.068
Positive	57 (17.1%)	11 (42.3%)	46 (14.9%)				
Negative	277 (82.9%)	15 (57.7%)	262 (85.1%)				
Postoperative immunohistochemical expressions of Ki-67				4.637 (1.460, 14.728)	0.005	–	–
≥70%	95 (28.4%)	13 (76.5%)	82 (41.2%)				
<70%	121 (36.2%)	4 (23.5%)	117 (58.8%)				
NA	118 (35.3%)	9	109				

ACIS, cervical adenocarcinoma in situ; AGC, atypical glandular cells; ASC-H, atypical squamous cells cannot exclude high-grade squamous intraepithelial lesions; ASC-US, atypical squamous cells of undetermined significance; CC, cervical cancer; CKC, cold knife conization; ECC, endocervical curettage; hrHPV, high-risk human papillomavirus; HSIL, high-grade squamous intraepithelial lesions; LEEP, loop electrosurgical excision procedure; LSIL, low-grade squamous intraepithelial lesion; NILM, negative for intraepithelial lesion and malignancy; SD, standard deviations; TZ, transformation zone. NA means missing data.

## Discussion

As the precursor to CAC, the incidence of ACIS is increasing, and the onset age is trending younger [16]. Hysterectomy is the recommended treatment for ACIS due to discontinuous lesions and the difficulty in determining the depth of invasion [9]. However, many women with ACIS have not completed childbearing and prefer cervical conization as an alternative. Additionally, some patients who have completed childbearing still wish to preserve their fertility. Therefore, fertility-sparing techniques such as cervical conization have emerged as alternative treatments for women with ACIS who wish to preserve their fertility. A retrospective study found that conization and sentinel node mapping was a safe and effective treatment option for early-stage CC, minimizing invasiveness while maintaining the oncological safety of more extensive procedures [17]. It is important to note that cervical conization, including CKC and LEEP, can lead to potential adverse postoperative outcomes, such as residual lesions and recurrence. Currently, only a few studies have investigated the risk factors associated with poor postoperative outcomes of this relatively conservative treatment [18]. Therefore, we conducted this study to explore the risk factors associated with positive surgical margins, residual lesions, and pathological upgrading after cervical conization in patients with ACIS, and to evaluate the effectiveness and safety of this conservative treatment approach.

In this study, the rate of positive surgical margins was 17.1%, which is lower than the 20.0%−55.6% reported in previous studies [19]. This discrepancy may stem from variations in the criteria used to define positive surgical margins across different studies. Additionally, some studies have explored the rate of positive surgical margins in patients undergoing CKC and LEEP, respectively [20]. Traditionally, CKC is preferred over LEEP because the former can remove an intact specimen without cauterization artifacts [4]. Montz et al. reported that large loop excision of the transformation zone (LLETZ) had significant diagnostic and therapeutic limitations, as it can lead to inadequate interpretation of pathological specimens and surgical margins [21]. Furthermore, Wang et al. indicated that LEEP was associated with increased tissue fragmentation and a higher rate of uninterpretable surgical margins [22]. In our study, we observed no uninterpretable margins in either LEEP or CKC. Additionally, we found no statistically significant differences in positive surgical margins, residual lesions, and pathological upgrading between LEEP and CKC, which is consistent with previous research [22]. Notably, several studies have focused on carbon dioxide (CO_2_) laser conization as a significant advancement in this technology. Ferrari et al. conducted a study involving 1,270 women who underwent either CO_2_ laser conization or CKC to compare the efficacy of these two methods in treating preinvasive lesions of the cervix [23]. They found a lower rate of positive surgical margins in the CO_2_ laser conization group, while the likelihood of positive endocervical or deep margins was similar in cases of incidental diagnosis of CC. Among the cases of incidental CC diagnosis, adenocarcinoma presented a higher risk of positive endocervical or deep margins compared to squamous carcinoma, regardless of the conization technique used. Residual lesions in unresected tissue after cervical conization are considered sources of disease recurrence [24]. The probability of residual lesions was significantly higher in patients with a positive endocervical margin compared to those with a positive ectocervical margin [25]. In this study, the rate of residual lesions was 53.5%, with the previously reported overall risk of residual ACIS ranging from 14.1% to 55.0% [24]. Regarding postoperative pathological upgrading associated with poor prognosis, we found a rate of 7.8%. Jiang et al. reported a rate of 6.0%, while ElMasri et al. reported 12.0% [26,27]. Various factors, such as geographic location, follow-up duration, and patient compliance, may influence the rate of pathological upgrading.

In this study, a preoperative positive result from ECC was identified as an independent risk factor for positive surgical margins, residual lesions, and pathological upgrading after cervical conization in patients with ACIS. ACIS, often originating in the TZ of the cervix, can also occur higher in the endocervical canal, affecting the deeper parts of the endocervical clefts [9]. Additionally, 10.0% to 15.0% of cases exhibit multifocal disease, characterized by foci of ACIS separated by at least 2 mm of normal mucosa [28]. Therefore, ECC, which assesses whether the lesion extends into the cervical canal, is an effective method for predicting adverse outcomes following CKC or LEEP. If the preoperative histopathological results of ECC are positive, deeper excisions may be necessary to achieve clear margins and favorable outcomes. Although we achieved an average conization depth of 2.2 cm in these patients, it is clear that this depth is insufficient. To effectively minimize the risk of positive surgical margins, the resection depth should exceed 2.2 cm. Our study found that the patients were primarily in the 30–40 age group with a mean age of 39.5 years, similar to findings by Bruno et al. in 2024 [29]. This may indicate that the lifestyle of women of childbearing age, including factors such as frequent sexual activity and smoking, increases their susceptibility to the disease [16]. Additionally, as evidence accumulates that diet is a modifiable risk factor for several cancers, a study has found that various micronutrients may play a protective role against CC by intervening at different stages of the natural history of HPV infection, the development of cervical dysplasia, and the progression to invasive disease [30]. We also found that being over 45 years old was an independent risk factor for positive surgical margins. Bartin et al. reached the same conclusion through a multicenter retrospective study across seven French centers [31]. Due to the atrophy of the TZ and cervical canal in older patients, complete resection is relatively difficult, making them more likely to experience adverse postoperative outcomes such as positive surgical margins [9]. Our study found that for patients older than 45 years, the depth of conization should exceed 2.1 cm to minimize the risk of positive surgical margins. Additionally, our study reported that patients with more than two ACIS lesions were more likely to have positive surgical margins after conization, consistent with findings by Costales et al. [32]. The multifocal nature and discontinuity of the lesions make complete resection challenging, thereby increasing the likelihood of adverse postoperative outcomes like positive surgical margins. Therefore, patients with multifocal lesions should undergo adequate resection (>2.2 cm) and receive enhanced postoperative follow-up and management. Regarding the number of pregnancies and births, we found that patients with more than one childbirth experience might be more susceptible to residual lesions. Some researchers believe that multiple deliveries may lead to cervical damage, disrupt immune system functions, diminish resistance to external adverse factors, and contribute to serious invasive diseases [33]. Liu et al. found 50.6% of patients with positive surgical margins had residual lesions [24]. In this study, we found that the risk of residual lesions in ACIS was 87.7% when surgical margins were positive. Similar to previous studies, a positive surgical margin is an independent risk factor for residual lesions in this study [25]. Some studies have confirmed that the recurrence risk of ACIS is only 2.6% with negative margins but rises to 19.0% when margins are positive, indicating that margin status is a predictor of residual, recurrent, and progressive disease [3]. In our study, the residual lesion rate in patients with negative surgical margins was 46.6%, which is higher than that reported in other recent studies. This discrepancy may arise from a combination of several factors. First, our follow-up process was relatively rigorous and strictly adhered to the three-step diagnostic principle for cervical lesions, resulting in a low rate of missed diagnoses. Second, in our study, residual lesions included not only ACIS but also patients with HSIL. Third, although the widely accepted definition for the time frame of residual lesions is six months, the possibility of recurrence or new occurrences during this period cannot be overlooked. Therefore, it is likely that these three factors contribute to the relatively high residual lesion rate observed in our study, highlighting the need for increased attention to these aspects in future research.

In addition to clinical symptoms and forms of contraception, our study found that there was no significant association between preoperative cervical cytology and HPV testing results, TZ types and diagnoses under colposcopy, the procedure for cervical conization, and poor postoperative outcomes. This may be explained by the commonality of hrHPV infection among patients with ACIS, without distinguishing between different types of HPV [34]. Bogani et al. observed that patients with persistent HPV at 6 months post-primary conization had a 7.5% risk of HSIL recurrence, and those with persistence at 12 months had a risk of 13.1%; however, persistence beyond 12 months did not correlate with an increased risk of recurrence [35]. With the growing prevalence of HPV vaccination, a multicenter retrospective study emphasized the potential benefits of administering the vaccine to protect against lower genital tract dysplasia following hysterectomy for HSIL and early-stage CC [36]. Notably, managing HPV lesions remains a global challenge; thus, providing adequate and tailored treatment, especially for high-risk patients, is crucial for improving prognosis [37]. Furthermore, inadequate sampling, the location of ACIS within the cervical canal, and the difficulty in identifying relatively uncommon pathology all contribute to the reduced sensitivity in detecting complete glandular lesions in cervical cytology [38]. Particularly, sampling deep lesions is challenging because colposcopic sampling primarily focuses on visible lesions [39]. Additionally, over 50% of ACIS cases are associated with squamous lesions, which may obscure glandular epithelial lesions and increase the misdiagnosis rate. Ki-67, a proliferation marker expressed in the stratified squamous epithelium of cervical intraepithelial neoplasia lesions, correlates with the degree of disordered maturation [40]. By combining previous studies with the discontinuous data on Ki-67 collected in this research, we established a threshold of 70% [41–43]. However, due to significant missing data on diagnoses under colposcopy and expressions of Ki-67 in our study, these factors cannot be conclusively linked to poor postoperative outcomes. Numerous studies have found that both LEEP and CKC are safe and effective for the conservative treatment of ACIS, with similar adverse outcomes, consistent with our findings [26,44]. Although cervical conization for ACIS is a technique used to preserve fertility, it may still affect pregnancy outcomes. For example, a study identified the histological type of ACIS and CC as risk factors for preterm delivery following conization [45]. Nevertheless, this procedure remains a crucial treatment option for patients with ACIS who wish to maintain their fertility. Future research is expected to focus on this patient population, enabling more comprehensive studies and the development of treatment methods that minimize the impact on pregnancy outcomes.

Although we analyzed the risk factors associated with adverse outcomes after CKC or LEEP in patients with ACIS, our study has several limitations that should not be overlooked. Firstly, it is important to note that there may be a correlation between patient age and the number of births, which could significantly impact calculations. Secondly, inconsistencies in HPV testing methods make it difficult to determine the specific type of HPV infection. Finally, the follow-up period was relatively short, as many patients were not followed up on time after six months.

## Conclusion

This study found that positive ECC results were independent risk factors for positive surgical margins, residual lesions, and pathological upgrading after cervical conization in patients with ACIS. Moreover, patients with ACIS who were over 45 years old and had multiple lesions were more likely to have positive surgical margins following CKC or LEEP. Multiple births and positive surgical margins increased the risk of residual lesions. For these patients, it is crucial not only to focus on completely removing the lesion but also to enhance postoperative follow-up to prevent disease recurrence or progression. Additionally, in treating ACIS, especially for women with fertility needs, it is essential to consider a combination of factors including preoperative histopathological results of ECC, age, and the number of ACIS lesions to develop personalized treatment strategies. We hope this study will promote more comprehensive research, leading to the development of safer, more effective, and individualized treatments for patients with ACIS.

## Supporting information

S1 File Anonymous data. (ZIP)
